# A Possible Mobile Health Solution in Orthopedics and Trauma Surgery: Development Protocol and User Evaluation of the Ankle Joint App

**DOI:** 10.2196/16403

**Published:** 2020-02-26

**Authors:** Florian Dittrich, David Alexander Back, Anna Katharina Harren, Marcus Jäger, Stefan Landgraeber, Felix Reinecke, Sascha Beck

**Affiliations:** 1 Department for Orthopaedics and Orthopaedic Surgery Saarland University Medical Center and Saarland University Faculty of Medicine Homburg Germany; 2 Clinic of Traumatology and Orthopedics Bundeswehr Hospital Berlin Berlin Germany; 3 Department of Plastic, Reconstructive & Aesthetic Surgery Specialized Clinic Hornheide Münster Germany; 4 Department of Orthopaedics, Trauma and Recontructive Surgery, St. Marien Hospital Mülheim and Chair of Orthopaedics and Trauma Surgery University of Duisburg-Essen Essen Germany; 5 Clinic of Trauma, Hand and Reconstructive Surgery University Hospital Essen Essen Germany; 6 Sportsclinic Hellersen Lüdenscheid Germany

**Keywords:** smartphone, ankle sprain, rehabilitation, self-care, mHealth, mobile phone

## Abstract

**Background:**

Ankle sprains are one of the most frequent sports injuries. With respect to the high prevalence of ankle ligament injuries and patients’ young age, optimizing treatment and rehabilitation is mandatory to prevent future complications such as chronic ankle instability or osteoarthritis.

**Objective:**

In modern times, an increasing amount of smartphone usage in patient care is evident. Studies investigating mobile health (mHealth)–based rehabilitation programs after ankle sprains are rare. The aim of this study was to expose any issues present in the development process of a medical app as well as associated risks and chances.

**Methods:**

The development process of the Ankle Joint App was defined in chronological order using a protocol. The app’s quality was evaluated using the (user) German Mobile App Rating Scale (MARS-G) by voluntary foot and ankle surgeons (n=20) and voluntary athletes (n=20).

**Results:**

A multidisciplinary development team built a hybrid app with a corresponding backend structure. The app’s content provides actual medical literature, training videos, and a log function. Excellent interrater reliability (interrater reliability=0.92; 95% CI 0.86-0.96) was obtained. The mean overall score for the Ankle Joint App was 4.4 (SD 0.5). The mean subjective quality scores were 3.6 (surgeons: SD 0.7) and 3.8 (athletes: SD 0.5). Behavioral change had mean scores of 4.1 (surgeons: SD 0.7) and 4.3 (athletes: SD 0.7). The medical gain value, rated by the surgeons only, was 3.9 (SD 0.6).

**Conclusions:**

The data obtained demonstrate that mHealth-based rehabilitation programs might be a useful tool for patient education and collection of personal data. The achieved (user) MARS-G scores support a high quality of the tested app. Medical app development with an a priori defined target group and a precisely intended purpose, in a multidisciplinary team, is highly promising. Follow-up studies are required to obtain funded evidence for the ankle joints app’s effects on economical and medical aspects in comparison with established nondigital therapy paths.

## Introduction

### Background

An ankle sprain is one of the most frequent injuries, with an incidence of 1:10,000 individuals per day in amateur and high-performance sports in the United States [1]. With respect to the high prevalence of ankle ligament injuries and patients’ young age, optimizing aftercare and rehabilitation is mandatory [2]. Moreover, the economic burden of ankle sprains is enormous [3]. High medical, physiotherapeutic, and lost productivity costs burden health care systems and create the need for new, efficient diagnostic and therapeutic solutions [4].

To prevent long-term complications, complex ligament injuries and recurrent ankle sprains with progression to chronic ankle instability (CAI) have to be recognized. The development of ankle osteoarthritis (OA) as a long-term consequence of CAI was first shown by Harrington et al [5] in 1979. Following an ankle ligament injury, posttraumatic muscular insufficiency [6,7] and ankle OA were observed in 13% of the cases [8]. Therefore, the adequate and consequent treatment of an ankle sprain might prevent CAI and OA.

Nowadays, early functional treatment is considered the gold standard for the lateral ligament lesion of the ankle [2,9]. The latest national guideline published by the German Orthopedic Foot and Ankle Society (Deutsche Assoziation für Fuß und Sprunggelenk eV, DAF) also recommends a conservative approach to acute ligament tears of the lateral ankle joint [10].

In times of digitalization and emerging technologies, smartphones are regularly used to accomplish everyday tasks, such as Web-based banking and communication *via* messenger or email, and penetrate rapidly into more and more areas of life [11]. The portability and omnipresent accessibility of smartphones enable usage anywhere and anytime [12]. In general, the growing implementation of smartphones as a transfer media in medical context is evident [13].

It has already been shown that the patients’ acceptance is given for collecting personalized health-relevant data *via* software apps, to share these with their peers or the medical staff [14]. Moreover, mobile short message service text messages and apps can have a positive impact on the posttraumatic outcome by showing increased adherence to medications and protocols, improved clinic attendance, and decreased readmission rates and emergency room visits [15].

However, the implementation and use of mobile health (mHealth) in medical care, especially in the fields of orthopedics and trauma surgery, can still be regarded to be in an early stage. So far, only 13 serious medical apps in orthopedics and trauma surgery have been identified for regular use in outpatient and inpatient medical care in German-speaking countries [16]. In a survey among German orthopedic and trauma surgeons, the *Ankle Joint App* (*Sprunggelenks-App*, Mediploy GmbH, Langenfeld, Germany) was shown to be frequently chosen, although the medical usage rate was still very low at 2.3% [17].

Studies investigating mHealth-based diagnostics [18] or rehabilitation programs after lateral ankle sprains already exist, for example, in the Netherlands (app: *Strengthen your Ankle*) [19], where a positive influence on medical and economic aspects could be demonstrated [20-22]. To date, the application of posttraumatic mHealth solutions after ankle sprains has not been investigated in Germany.

### Objective

To address this gap, this work outlines the methodology to develop and design an app for patient education as well as prevention and identification of CAI after ankle sprains (*Ankle Joint App*). The publication of an app development process might be the basis for future mHealth solutions to improve patient care.

The app’s content, usability, and styling were evaluated by German orthopedic or trauma surgeons and athletes who suffered from ankle sprain.

## Methods

### Development Protocol

#### Basic App Conception

A multidisciplinary team was involved in the development of the *ankle joint app*. The team members comprised 2 orthopedic and trauma surgeons (FD and SB), a physiotherapist, a lawyer, and a software and Web developer. Before programming the app, some general aspects regarding the software structure, design, and content had to be considered. At an early stage, the target group and the intended purpose needed to be defined precisely to clarify whether the app had to be defined as a medical device and therefore had to be regulated by medical products law [23].

#### Technical Specifications

The *ankle joint app* was developed using *React Native* (Facebook Inc) technology. *React Native* is a *Javascript*-based framework for software developers, building cross-platform mobile apps for Android or iOS devices. The framework features built-in components and application programming interfaces, which are essential for developing innovative and user-friendly mobile apps [24].

The backend server runs on a Web app based on the *Hypertext Preprocessor* framework *Symfony* and meets actual software security guidelines. Any data exchange between the backend server and the app runs via *Secure Sockets Layer* secured connection. All server structures are located in Germany. Patient-related data remain strictly on the mobile device.

#### Texts and Videos

The *ankle joint app* is based on the latest national guidelines published by the German Orthopedic Foot and Ankle Society (DAF) and related medical literature. The content is written in German. All relevant references are stored in the app and are hyperlinked to the primary source to facilitate search for the user. Special efforts have been made to ensure that the information communicated is short, clear, and easy to understand. To explain medical terms comprehensibly, a glossary function has been integrated to avoid overloaded main text pages. By answering frequently asked questions, personal data are collected and made available to the user at some key points. Thus, the content adapts to the individual healing process constantly.

**Figure 1:**
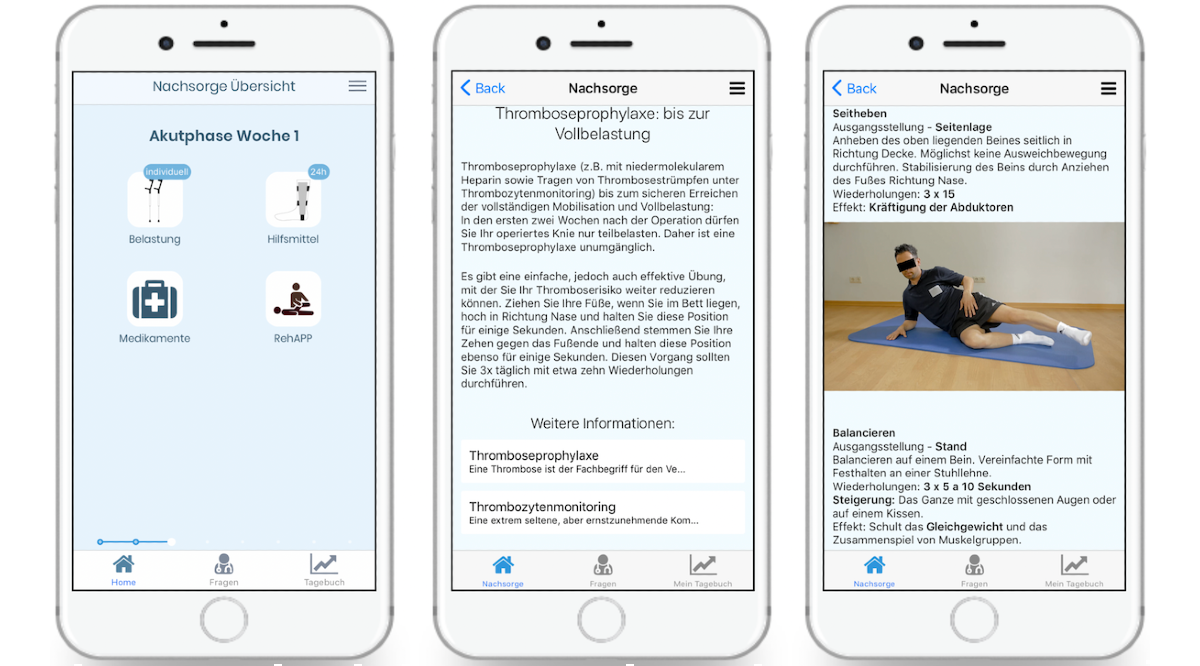
App screen view: (a) timeline-based aftercare plan, (b) information, and (c) training videos.

With the cooperation of a physiotherapist and considering the current research data, a training program was created, which can be carried out without special equipment. In addition to giving some general information, for example, the PRICE-rule (P=protection, R=rest, I=ice, C=compression, and E=elevation) or the activation of the muscle-vein pump in the acute stage, patients are also provided with short video clips in the later stages (Figure 1). A total of 15 successive built-up exercises were made available to patients via the app. A special focus in this training circle was placed on early functional mobilization and proprioceptive training to prevent CAI.

#### Patient-Generated Data and Log Function

**Figure 2:**
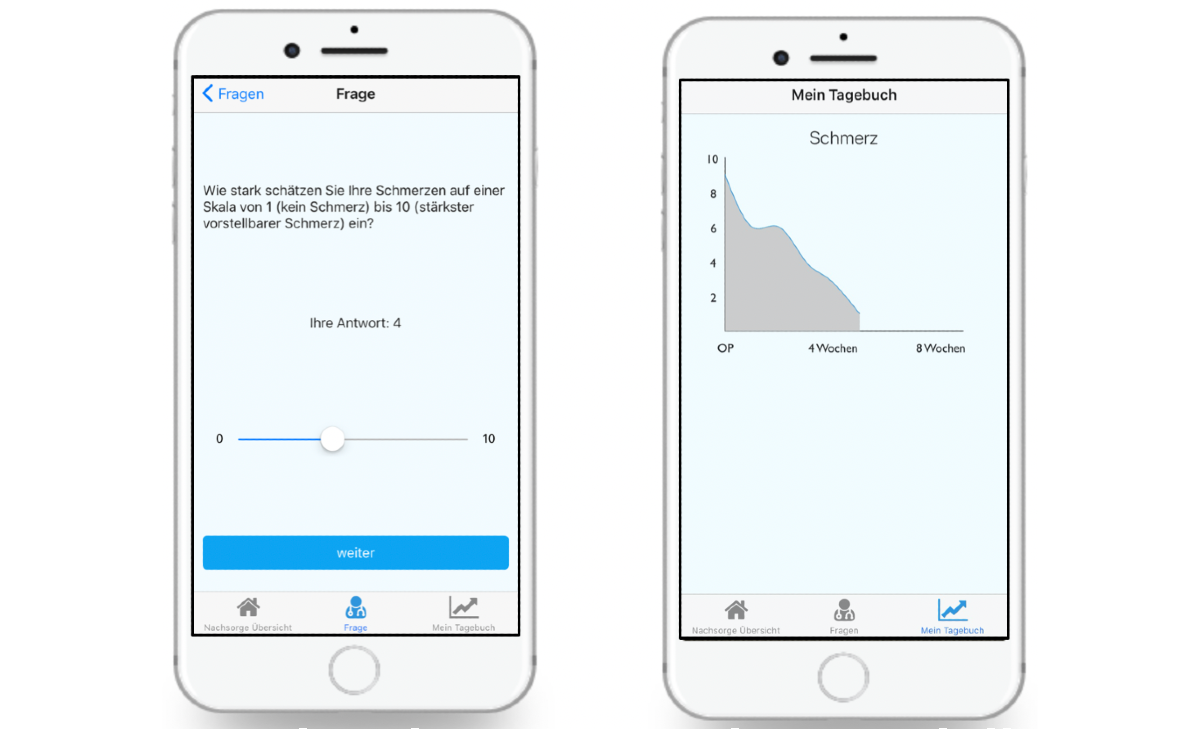
App screen view: (a) collection of patient-related data and (b) log function of patient-related data.

Initially, to start the timeline, the timepoint of the injury has to be defined. In addition, the user is asked to evaluate whether it was the first or the second event of an ankle sprain. Moreover, the kind of trauma and prescribed aids are requested. At regular intervals, patients are asked questions via push messages about their current level of pain, using the visual analog scale; feeling of instability; and load-bearing capacity. The collected patient-related data are presented to the patient in an understandable graphical form in the diary function (Figure 2).

#### German Cumberland Ankle

The Cumberland Ankle Instability Tool (CAIT) was developed for measuring the severity of functional ankle instability [25]. Using a well-established 9-item 30-point scale, the CAIT shows an adequate correlation to performance tests. It is a valid and reliable instrument for assessing CAI [26-28]. The minimal detectable change, as well as the minimal clinical important difference, lies at ≥3 points [29]. We assessed the status of CAI using the validated German CAIT in a digital form for the first time [30,31]. The CAIT was surveyed on day 56 after trauma. A score of <25 indicates CAI, and the app user is informed that additional diagnostics are recommended [32].

#### Styling, Design, and Testing

Special attention was paid to the development of an intuitive and user-friendly interface. To allow elderly patients to use the app, an onboarding feature was established to explain the main functions and interactions. Milestones in rehabilitation were presented graphically in a timeline to ensure clarity for patients about the progress of their rehabilitation. Information regarding the rehabilitation process was structured logically and linked to icons with a recognition factor.

After the app had been developed, an *alpha* test was carried out by the development team using the software *TestFlight* (Apple Inc). The following *beta* test was performed on persons who had already undergone an ankle distortion trauma. Some technical, content-related, and interactional improvements were made as a consequence to the test feedback under controlled conditions.

### App Quality Testing

#### Study Design and Sample

The app was evaluated by 20 German orthopedic and trauma surgeons with a special interest in foot and ankle surgery as well as 20 athletes. All the physicians and athletes included were familiar with smartphone devices and used apps on a daily basis. The involved athletes already sustained an acute ankle sprain in their past sporting career. The study was conducted between June 2019 and August 2019. The link for the digital questionnaire on a *Google Docs* (Google LLC) platform was sent to the participants by email or Quick Response code scanning. The email addresses of the physicians were generated manually via the home pages of clinics or via established email distribution lists. The athletes were screened in local badminton or boxing clubs (FC Langenfeld, VFL Bochum, and Lanna martial arts Bochum).

#### German Mobile App Rating Scale

The MARS rating is a well-established assessment scale for medical app quality. It was developed for professionals, and it includes the sections classification, objective app quality, subjective app quality, and a modifiable app-specific section. MARS items are scored using a 5-point *Likert* scale (1=inadequate, 2=poor, 3=acceptable, 4=good, and 5=excellent). The objective app quality section includes 19 items divided into 4 subscales—*engagement*, *functionality*, *esthetics*, and *information quality*—and a separate *subjective app quality* section*.* The *subjective app quality* section contains four items evaluating the user’s overall satisfaction.

Calculating the mean scores of the engagement, functionality, esthetics, and information quality objective subscales, as well as an overall mean app quality, total score is how the MARS is scored. Mean scores instead of total scores are used because items can be rated as *not applicable*. The subjective quality items can be scored separately as a mean subjective quality score [33].

The English MARS version’s sections were extended in the MARS-G by an additional section focusing on the *medical gain* of an app. The 5 subscales and the overall score determine the app’s quality [34]. All surgeons watched the associated MARS-G instructional video on how to use the MARS-G scale before rating in case of doubt [35].

### Data Analysis

The analog (user) MARS-G was converted into a digital questionnaire on a *Google Docs* platform (Google LLC). Data were saved and then transferred into an *Excel* table (Microsoft Corp). Descriptive statistics were calculated for all items. The intraclass correlation coefficient (ICC) was calculated among the reviewers. We selected an individual absolute agreement ICC (AA-ICC) for a two-way mixed model on the basis of ICC guidelines by Shrout and Fleiss [36]. All statistical analyses were conducted using SPSS (version 25, IBM Corp).

## Results

### Participants

A total of 20 foot and ankle surgeons as well as 20 athletes who suffered an ankle sprain took part in the app rating, which is equivalent to a response rate of 65% (20/31) for the surgeons and 44% (20/46) for the athletes. Excellent interrater reliabilities (two-way mixed model AA-ICC=0.92; 95% CI 0.86-0.96 for surgeons and athletes) were shown following the guidelines for ICC interpretation established by Koo et al [37]. The surgeons’ group comprised 20% (4/20) Android and 80% (16/20) iOS users, and the athletes’ group comprised 59% (10/17) Android and 41% (7/17) iOS users.

### German Mobile App Rating Scale

**Figure 3:**
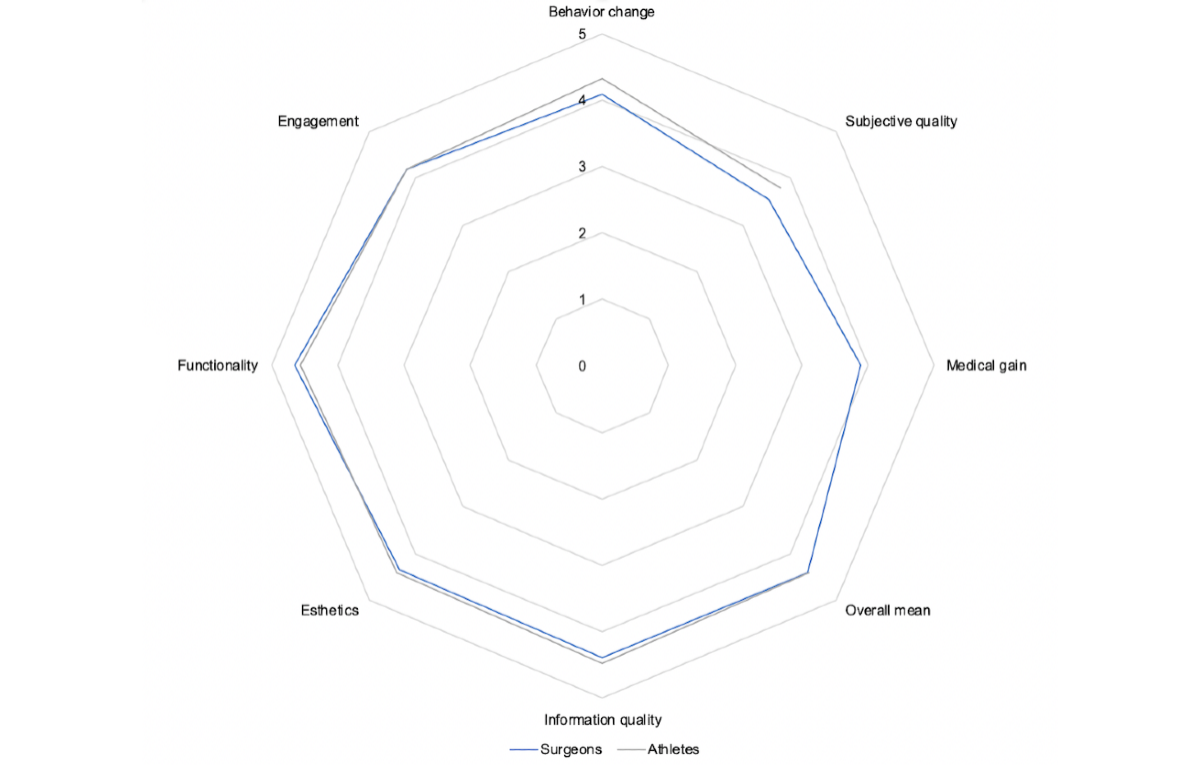
Mean scores of the (user) German Mobile App Rating Scale for the Ankle Joint App (surgeons: n=20 and athletes: n=20).

The mean overall score for the *Ankle Joint App* was 4.4 (SD 0.5), rated by both surgeons and athletes. It was derived from the mean scores on app *functionality*, *engagement*, *esthetics*, and *information quality* (Figure 3). The mean subjective quality scores were 3.6 (surgeons: SD 0.7) and 3.8 (athletes: SD 0.5). The section *behavioral change*, which included an assessment of the perceived impacts on disease-related knowledge, attitude, awareness, and behavior, had mean scores of 4.1 (surgeons: SD 0.7) and 4.3 (athletes: SD 0.7). The *medical gain,* rated by the surgeons only, was 3.9 (SD 0.6; Table 1, Figure 3).

**Table 1:** Detailed results of the (user) German Mobile App Rating Scale.

Subscale		MARS-G^a^ surgeons			uMARS-G^b^ athletes		
		Minimum	Maximum	Mean (SD)	Minimum	Maximum	Mean (SD)
**Engagement**							
	Entertainment	3	5	4.4 (0.9)	3	5	4.1 (0.7)
	Interest	3	5	4.3 (0.9)	2	5	4.4 (0.9)
	Customization	2	5	3.8 (0.8)	2	5	3.6 (0.9)
	Interactivity	3	5	4.2 (0.7)	2	5	4.3 (0.9)
	Target group	4	5	4.6 (0.5)	4	5	4.6 (0.5)
**Functionality**							
	Performance	4	5	5.0 (0.2)	3	5	4.8 (0.6)
	Usability	4	5	4.6 (0.5)	3	5	4.6 (0.6)
	Navigation	4	5	4.6 (0.5)	3	5	4.5 (0.7)
	Gestural design	4	5	4.5 (0.5)	4	5	4.5 (0.5)
**Esthetics**							
	Layout	3	5	4.4 (0.8)	3	5	4.5 (0.6)
	Graphics	4	5	4.6 (0.5)	3	5	4.5 (0.6)
	Visual appeal	3	5	4.1 (0.7)	3	5	4.3 (0.7)
**Information**							
	Accuracy of app description (in app store)	3	5	4.8 (0.5)	—^c^	—	—
	Goals	3	5	4.3 (0.6)	—	—	—
	Quality of information	3	5	4.5 (0.8)	4	5	4.7 (0.5)
	Quantity of information	4	5	4.8 (0.4)	3	5	4.9 (0.5)
	Visual information	4	5	4.3 (0.5)	3	5	4.5 (0.6)
	Credibility	3	5	4.0 (0.9)	2	5	3.8 (1.0)
	Evidence base	2	5	4.0 (1.1)	—	—	—
**Medical gain**							
	Gain for patients	3	5	4.3 (0.8)	—	—	—
	Gain for physicians	2	5	3.8 (0.9)	—	—	—
	Risks, side and adverse effects	3	5	4.5 (0.6)	—	—	—
	Transferability into routine care	2	5	3.3 (1.0)	—	—	—
**Subjective quality**							
	Would you recommend this app to people who might benefit from it?	3	5	4.4 (0.9)	3	5	4.3 (0.7)
	How many times do you think you would use this app in the next 12 months if it was relevant to you?	1	5	3.2 (1.2)	2	5	3.9 (0.7)
	Would you pay for this app?	1	3	2.0 (0.7)	1	3	2.4 (0.8)
	What is your overall star rating of the app?	4	5	4.6 (0.5)	3	5	4.6 (0.6)
**Behavior**							
	Awareness	2	5	4.5 (0.8)	3	5	4.6 (0.6)
	Knowledge	3	5	4.6 (0.6)	3	5	4.8 (0.6)
	Attitudes	2	5	3.7 (0.9)	3	5	4.3 (0.7)
	Intention to change	3	5	3.9 (0.8)	4	5	4.4 (0.5)
	Help seeking	2	5	4.1 (1.0)	2	5	3.8 (1.0)
	Behavior change	2	5	4.0 (0.9)	3	5	4.3 (0.8)

^a^MARS-G: German Mobile App Rating Scale.

^b^uMARS-G: (user) German Mobile App Rating Scale.

^c^Not applicable.

## Discussion

### Principal Findings

Our research using the *Ankle Joint App* demonstrates that mHealth-based rehabilitation programs might be an adequate and innovative tool for patient education, prevention, and collection of personal data.

The achieved (user) MARS-G scores prove the app’s quality from a professional and user point of view, demonstrating a comparatively high overall mean (user) MARS-G value [33]. The highest scores were reached in the functionality section for both surgeons and athletes. In accordance with a recent survey among orthopedic and trauma surgeons, intuitive usability was considered the most important factor for the regular use and quality of an app. The integration of complex functions in an intuitive lean and secure user interface poses a great challenge to the development team. Moreover, the development of an intuitive *frontend* is complex and involves high development costs and test phases [38]. Multifunctional apps, for example, in the field of diabetes mellitus type II therapy for patients over 50 years showed limited usability with negative effects on compliance and therapy outcomes. Apps with basic functions provide enhanced usability [39], but the limitation of software features affects the app’s functionality. To address this divergence, trial runs with specific target groups and permanent reevaluation of the initial concept are mandatory during the app’s development process.

As a first step, when developing an app, the target group and the intended purpose have to be defined precisely. The *Ankle Joint App* was especially designed for young and active patients to optimize conservative rehabilitation following an acute ankle sprain without osseous lesions. With respect to this, the differences in evaluating the medical gain for physicians and patients, with individual requirements in their rehabilitation episode [40], can be explained.

Customization seems to be important to the target group and might be improved in our app. We believe that medical apps have to be adaptable not only to the specific users’ requirements but also to the varying hospital standards. The aspects to be considered in the development of medical apps are the limited areas of application in combination with varying standards of treatment, both national and international, and the legal and medical aspects of an app with regard to liability and data protection [21]. These aspects represent a challenge for financing the complex development and maintenance of an app, as the 10 most popular apps ranked by the number of users in Germany in 2018 were all available for free download [41].

Considering recent data scandals, which led to a fundamental distrust of apps that might be implemented in the context of *Big Data*, the secure and transparent collection of medical personal data can be challenging [42,43]. For this reason, we decided to store personal data exclusively on the mobile device to avoid cloud upload. In general, dichotomous scenarios about data exchange between patients and medical staff are possible. The recovery progress could be displayed analogously on the patient’s smartphone in the event of a doctor’s appointment (eg, CAIT). Alternatively, an upload of data into a secure cloud system can be taken into consideration [44]. In the event of deviations from the expected progression of the disease, patients could be informed about and provided with medical expertise more rapidly. Moreover, the collection of validated scores and surveys might be relevant for academic research.

In contrast to these positive effects, app users and providers (physicians and medical staff) should keep in mind that the collection and processing of personal data also represent cornerstones of app financing. This could lead to potential conflicts of interest as the collected data represent an immense value, for example, for the provision of personalized advertising [45]. Before downloading an app, the financing, development process, and data flow have to be completely and plausibly depicted by the publisher. Therefore, transparent and appropriate app store descriptions, data protection regulations, and terms and conditions of use are of utmost importance [46].

This study has some limitations. The evaluation of the app’s quality was carried out within a theoretical framework; thus, it only reflects its use to a limited extent in daily clinical practice. It has to be mentioned that the app was only evaluated by a relatively small number of users (patients) for a short period of 3 months. Thus, data on compliance, demographics, and usage behavior are hardly representative. Moreover, the economic aspects of the development process and app costs per user were not taken into account. In addition, the response rate was moderate, which might lead to a bias toward users with high digital affinity. Comparative randomized control trial studies are required to gain funded evidence on the app’s positive effects on patient education and treatment progress in comparison with established nondigital therapy paths to prevent CAI and reach a final scientific conclusion; this has to be addressed by future studies.

Nowadays, in many countries, an increasing number of patients visiting emergency units with minor complaints can be registered. Often, the treatment of these patients is time and staff consuming, compromising the medical attention of more severely injured individuals. This overcrowding may lead to negative consequences to the patients’ safety and their outcome [47-49]. Given the increasing workload, physicians are dissatisfied with highly time-consuming procedures, for example, electronic patient recording in the emergency department [50,51]. Apps might be used by the emergency staff to easily create and recommend digitally customized aftercare plans.

Moreover, the established discharge letter contains medical terminology, which offers very little benefit to a self-determined, competent patient. This practice does not meet the requirements of adequate patient involvement in the treatment process [52]. Improving patient education and optimizing the communication structures via apps on mobile devices have the potential to solve these issues. Individually designed and supervised aftercare treatments showed better outcomes [53]. The *Ankle Joint App* has a modular design and might be transferred to a wide range of aftercare treatments.

In contrast to the great potential of standardized medical app usage, there are also risks. However, medical resources and health care have to be distributed equally for everyone on the basis of moral and ethical obligations. This is why medical app usage also entails a particular risk of disadvantaging groups with low health competence and a high risk of disease [39]. In particular, the elderly patient might be disadvantaged by the use of medical apps, because in 2014, only about 17% of the individuals over 65 years regularly used a smartphone [54]. As degenerative diseases represent an important pillar of orthopedic and trauma surgery expertise, special attention has to be paid on the app development for these *newcomers* and their requirements in the future. Particularly in the area of app usability, the requirements of elder generations have to be addressed, for example, implementing an intuitive interface, a reading function, or a screen magnifier [55]. Self-endangerment because of incorrect app usage might occur, but the risk can be reduced by an *onboarding* function with an introduction of the app to new users and individual feedback mechanisms [56].

### Conclusions

Working in a multidisciplinary team, using a backend structure to modify the app’s content and using React Native, proved to be efficient in the development process of medical apps. The success was proven by reaching high overall mean MARS-G scores for the *Ankle Joint App* in surgeons and athletes. Data obtained suggest that an mHealth-based rehabilitation program might be a useful tool for patient education and collection of personal data. The achieved (user) MARS-G scores prove the tested app’s high quality. Medical app development with an a priori defined target group and a precisely intended purpose, in a multidisciplinary team, is highly promising.

## Abbreviations

AA-ICC: absolute agreement intraclass correlation coefficient
CAI: chronic ankle instability
CAIT: Cumberland Ankle Instability Tool
ICC: intraclass correlation coefficient
MARS-G: German Mobile App Rating Scale
mHealth: mobile health
OA: osteoarthritis
